# Antimicrobial Blue Light Reduces Human-Wound Pathogens’ Resistance to Tetracycline-Class Antibiotics in Biofilms

**DOI:** 10.3390/cells14030219

**Published:** 2025-02-04

**Authors:** Laisa Bonafim Negri, Sandeep Korupolu, William Farinelli, Alexis K. Jolly, Robert W. Redmond, Shifu Aggarwal, Laurence G. Rahme, Kristin H. Gilchrist, R. Rox Anderson, Jeffrey A. Gelfand

**Affiliations:** 1 Wellman Center for Photomedicine, Massachusetts General Hospital (MGH), Boston, MA 02114, USA; lnegri@mgh.harvard.edu (L.B.N.); skorupolu@mgh.harvard.edu (S.K.); bfarinelli@mgb.org (W.F.); rredmond@mgh.harvard.edu (R.W.R.); rranderson@mgh.harvard.edu (R.R.A.); 2 Vaccine & Immunotherapy Center, Division of Infectious Diseases, Massachusetts General Hospital (MGH), Boston, MA 02114, USA; 3 Department of Dermatology, Massachusetts General Hospital, Boston, MA 02114, USA; 4 Department of Dermatology, Harvard Medical School, Boston, MA 02114, USA; 5 School of Medicine, University of Edinburgh, Edinburgh EH16 4UX, UK; akjolly@mgh.harvard.edu; 6 Department of Surgery, Massachusetts General Hospital, Harvard Medical School, Boston, MA 02115, USA; saggarwal5@mgh.harvard.edu (S.A.); lgrahme@mgb.org (L.G.R.); 7 Department of Microbiology, Harvard Medical School, Boston, MA 02114, USA; 8 Shriners Hospitals for Children Boston, Boston, MA 02114, USA; 9 4D Bio3 Center for Biotechnology, Department of Radiology and Bioengineering, Uniformed Services University of the Health Sciences, Bethesda, MD 20817, USA; kristin.gilchrist.ctr@usuhs.edu; 10 The Geneva Foundation, Tacoma, WA 98402, USA; 11 Department of Medicine, Harvard Medical School, Boston, MA 02115, USA

**Keywords:** antimicrobial blue light, antimicrobial resistance, biofilms, tetracyclines, wound infection, phototherapy

## Abstract

Biofilms contribute to chronic infections and the development of antimicrobial resistance (AMR). We are developing an antimicrobial blue light (aBL) device to reduce bacterial bioburden in wounds and decrease reliance on systemic antibiotics. aBL induces the generation of reactive oxygen species (ROS) through photoexcitation of endogenous chromophores, causing bacterial damage and death. This study explores the combination of tetracyclines (TCs) with aBL for the treatment of biofilm infections in vitro. Tetracyclines (TCs), including second-generation minocycline (MC), doxycycline (DOCT), and third-generation agents omadacycline (OM) and tigecycline (TG), were evaluated for their ability to enhance bactericidal effects and ROS production during aBL treatment of abiotic biofilm. TCs were tested under dark conditions and with varying aBL light parameters against biofilms of methicillin-resistant *Staphylococcus aureus* (MRSA), *Pseudomonas aeruginosa* (PA), and *Escherichia coli* (*E. coli*). Results showed that TCs alone were ineffective against these biofilm cultures. However, when combined with aBL either before or after TC treatment, significant enhancement of microbicidal activity was observed. When the aBL is added before the TCs, there was equivalent bactericidal effect, indicating that TCs primary action against biofilms were not as photosensitizers. These findings suggest that aBL can significantly enhance the antimicrobial activity of TCs, potentially offering a new effective approach to treating biofilm-associated infections and combating AMR when aBL is applicable.

## 1. Background

Biofilms are found in many of the most serious and difficult to treat bacterial infections, with estimates as high as 80% of serious human infections being biofilm associated [1,2,3,4]. Biofilm-resident bacteria are dramatically more resistant to antibiotics than standard antimicrobial testing predicts, anywhere from 10 to 1000-fold more resistant than the same bacteria in planktonic culture [5,6]. Biofilms enable additional genotypic and phenotypic antibiotic resistance to occur [7]. The poor penetration of antibiotics into biofilms, the decrease in metabolic activity of many bacteria in biofilms, and the exchange of antimicrobial resistance elements between bacteria in the biofilm all provide perfect conditions for the further growth of antimicrobial resistance (AMR) [7].

We are confronting a future in which the most common medical problems could become high-risk/high-mortality events owing to infection with newly AMR organisms [8,9]. Reasons for increasing AMR and paucity of new antibiotic options are beyond the purview of this manuscript, but wounds, especially chronic wounds, are a significant contributor to development of antibiotic resistance [8,10]. Chronic wounds often become biofilm infections [7,8]. The most common wound isolate worldwide is *Staphylococcus aureus* (SA), and methicillin-resistant *Staphylococcus aureus* (MRSA) is the most common multidrug-resistant (MDR) organism worldwide. Investigators have found resistance genes to tetracyclines, beta-lactams and glycopeptides in permafrost samples that are over 30,000 years old (reviewed in [8,11]), now made worse by the ubiquitous and continued indiscriminate use of antibiotics in livestock and human medicine.

We are developing an antimicrobial blue light (aBL) device. The purpose is to accelerate the reduction in bacterial bioburden in wounds and enable reduced systemic antibiotic use for treatment of wound infections [12]. Intended for the treatment of wounds reachable by light, the aBL device is based on work by many others demonstrating the antibacterial applications of aBL [8,13,14,15,16,17]. Our own experiments have demonstrated that several logs of bacterial colony-forming units (CFU) in biofilms can be killed by aBL (>99.99%), thus potentially reducing the need for, or quantity of, systemic antibiotics to control a surface wound. Despite many years of investigation and many supportive small animal model publications, there are no licensed devices approved for treatment of wound infections by either the U.S. Food and Drug Administration (FDA) or the European Medicines Agency (EMA). Clinical studies of aBL have been largely anecdotal and poorly controlled.

ABL functions primarily by generating reactive oxygen species (ROS) upon interacting with photosensitive compounds, or chromophores, within microbial cells. Blue light, typically in the wavelength range of 400–470 nm, penetrates the cells and activates endogenous photosensitizers, such as porphyrins, which in turn produce ROS, including singlet oxygen and free radicals, within the bacteria. These reactive species lead to oxidative damage to essential bacterial components, such as proteins, lipids, and nucleic acids, ultimately disrupting the integrity of the microbial cell membrane and triggering cell death. Furthermore, aBL can break down biofilm structures, enhancing bacterial susceptibility to treatment. This mechanism underscores the promise of aBL as an innovative, non-invasive strategy for addressing antibiotic-resistant infections while minimizing damage to surrounding tissues and potentiating the antimicrobial activity of antibiotics [15]. Other forms of antimicrobial phototherapy, such as photodynamic therapy incorporating exogenous photosensitizer drugs with various wavelengths of light, may yet add to the antimicrobial therapeutic armamentarium [18], currently underutilized against infection.

The “dose” (fluence) of light is determined by the product of light irradiance (power density) and exposure time. A lower irradiance is preferable as it reduces heat. In this context, we explored the potential benefits of incorporating topical antibiotics with blue light treatment for biofilms, to potentially enable lower light doses while still avoiding systemic antibiotic use. This approach aligns with our goals of minimizing systemic antibiotics while speeding resolution, ensuring that total antibiotic administration remains low.

We chose to initially focus on the tetracyclines (TCs) because of their low cost, wide use, routine topical application, generally low toxicity, and the recent development of tetracyclines with expanded drug potency against a myriad of bacterial species if needed. The case for combining the tetracyclines with aBL is comprehensively reviewed [8,19]. However, very little of the extensive literature involving tetracyclines and aBL has examined this interaction in biofilms, having been conducted primarily in planktonic cultures. Yet, biofilms are the very form in which the bulk of human-wound infections, suitable for such light-enhanced treatment, actually exist [20].

*E.coli* was demonstrated to photo-incorporate tetracycline, photolabeling *E. coli* 30S ribosomes [8,21], suggesting that this contributed substantially to the drug’s pharmacologic activity. This was further studied by Hasan and Kahn [22], who demonstrated that irradiation of chlortetracycline by 389–404 nm light resulted in the generation of singlet oxygen, hypothesizing that this photodynamic mechanism was a cause of tetracycline photosensitivity and likely contributed to antibacterial activity. Further investigations at our institution demonstrated that tetracyclines, which can accumulate in bacterial ribosomes, could be photoactivated with blue light [20,23]. These observations were all made in planktonic cultures.

We examined the interaction of aBL with the commonly used second-generation as well as extended spectrum, third-generation tetracyclines. We screened them in in vitro biofilms to select the optimal all-around topical antibiotic applications to be used in combination with an aBL device to treat biofilm wound infections. We compared these light–drug interactions in biofilms with the antimicrobial activity of the same drugs in planktonic cultures of bacteria. All of this was performed with the intent of improving the treatment of biofilm wound infections by introducing aBL to the therapeutic equation, to hopefully reduce the overall need for systemic antibiotics. In our current experiments, we focused on the activity of four widely used tetracyclines: the second-generation antibiotics doxycycline (DOCT) and minocycline (MC), and the third-generation agents tigecycline (TG) and omadecycline (OM), with broader-spectrum activity.

## 2. Materials and Methods

### 2.1. Absorption UV–Vis Spectroscopy

Different TC concentrations were prepared in Phosphate-buffered saline (PBS) to establish a calibration curve. Absorbance spectra from 200 to 800 nm were obtained using an Evolution™ 300 UV–Vis Spectrophotometer (ThermoFisher Scientific, Waltham, MA, USA). The resulting calibration curve was used to calculate the molar absorption coefficient.

### 2.2. Chemicals

All TCs (doxycycline, minocycline, demeclocycline, omadacycline, and tigecycline) were obtained from Sigma-Aldrich (St. Louis, MO, USA). PBS and Brain-heart infusion broth (BHI) were sourced from Fisher Scientific (Waltham, MA, USA). Luria Broth (LB) media was purchased from Sigma-Aldrich. Stock solutions of TCs at 10 mg/mL were freshly prepared in PBS and subsequently diluted to the final concentration in PBS before use.

### 2.3. Blue Light Source and Light Measurements

For aBL irradiation, a light-emitting diode (LED; M405L4; Thorlabs, Newton, NJ, USA) with a peak emission at 410 nm and a full width at half maximum of 25 nm was used as the light source. The power density/irradiance (mW/cm^2^) was measured at the target surface using a PM100D power meter (Thorlabs, Newton, NJ, USA). Various power densities were tested: 50, 30, 15, and 10 mW/cm^2^. The dose of light (fluence or radiant exposure) was calculated using the formula:

Dose, or Fluence (J/cm^2^) = Irradiance or power density (W/cm^2^) × Exposure Time (seconds)

### 2.4. Bacterial Strains and Growth Conditions

The strains used in this study were all clinical isolates. *Methicillin-resistant* Staphylococcus aureus USA300 (MRSA) and *Escherichia coli* ATCC 25922 (*E. coli*) were obtained from the American Type Culture Collection (ATCC), while *Pseudomonas aeruginosa* UCBPP-PA14 (PA) was generously provided by Dr. Laurence Rahme, originally isolated from a patient with sepsis. All strains were routinely cultured on BHI or LB agar plates and incubated overnight at 37 °C in 5% CO_2_.

### 2.5. Minimum Inhibitory Concentrations (MICs) for TCs (DOCT, MC, TG, OM)

The MICs of TCs against various bacterial strains were determined using a standard broth microdilution assay, following the guidelines of the Clinical and Laboratory Standards Institute (LCSI) [24]. Briefly, a stock solution of TCs was prepared at 10 mg/mL in PBS. This stock solution was diluted to 2.048 mg/mL in BHI (for MRSA and *E. coli*) and LB (for PA), followed by serial dilutions down to 0.007 μg/mL in a 96-well plate. A 10 μL aliquot of stationary-phase bacterial culture (10^8^ CFU/mL) in BHI or LB broth was added to each well containing the TC dilutions. BHI or LB broth without any drug served as the control. The microplates were incubated at 37 °C for 24 h, and the minimum concentration of TCs that completely inhibited bacterial growth was recorded as the MIC [25].

### 2.6. Photoinactivation of Bacteria in Biofilm Culture by Tetracyclines (DOCT, MC, TG, OM), Followed by aBL

The bacterial suspension was cultured overnight in a shaking incubator using BHI or LB broth. Following growth, the cells were collected by centrifugation at 4000 rpm for 5 min and resuspended in PBS to a density of 10^8^ CFU/mL (OD_600_ = 0.1) and diluted for 10^6^ CFU/mL [26]. Biofilms were then grown in 96-well microtiter plates for 48 h, with media changes every 24 h. After 48 h, 200 μL of PBS with or without TCs at varying concentrations (0, 1, 2, and 4 μg/mL) was added to the wells, and the biofilms were incubated in the dark at room temperature for 83 min. Following incubation, biofilms treated with TCs and control biofilms were kept in the dark, while other biofilm groups were exposed to aBL at different power densities (50, 30, 15, and 10 mW/cm^2^) with constant radiant exposure of 250 J/cm^2^. After light exposure, the biofilms were washed twice with PBS, and 200 μL of PBS was added to each well. The bacterial biofilms were then harvested by scraping with a sterile pipette tip, and the contents of three wells per group were pooled into a 1.5 mL microcentrifuge tube. The pooled volume of 600 μL (from 3 wells) was sonicated for 5 min using a Branson 2510 Water Bath Sonicator (Marshall Scientific, LLC, Hampton NH, USA). CFU/mL was determined by performing 10-fold serial dilutions in PBS, plating on BHI or LB agar plates, and counting the colonies after overnight incubation at 37 °C. The experiments were conducted in triplicate [26].

### 2.7. Evaluation to Determine the Effect of Post-aBL Incubation Time of TCs on Subsequent Bacterial Viability

After 48 h, 200 μL of PBS, with or without TCs (4 μg/mL), was added to each well, and the biofilms were incubated in the dark at room temperature for 83 min. Following this, the biofilms treated with TCs and the controls were exposed to aBL at an intensity of 50 mW/cm^2^, yielding a total radiant exposure of 250 J/cm^2^. After light exposure, TCs in PBS were removed and replaced with either BHI alone or BHI supplemented with TCs (4 μg/mL). Bacterial biofilms were then harvested using the same method as described previously. The effect of blue light was assessed after incubation with either TCs or BHI alone at different time points: TCs + aBL without additional incubation; TCs + aBL followed by incubation with TCs for 24 h; and TCs + aBL followed by incubation with TCs for 48 h. As a control, the same experiment was conducted using BHI alone instead of BHI with TCs at the same time points. Additionally, the experiment was replicated under identical conditions without light irradiation, serving as a dark control for each treatment. CFU/mL was quantified through 10-fold serial dilutions in PBS, followed by plating on BHI and counting colonies after overnight incubation at 37 °C. All experiments were performed in triplicate.

### 2.8. Evaluation of Photosensitization Ability of TCs: Photoinactivation of MRSA in In Vitro Biofilms by aBL, Preceded or After Addition of TCs

The bacterial suspensions were grown overnight in 10 mL BHI broth at 37 °C and 200 rpm for up to 16 h. Then, the biofilms were grown for 48 h in 96-well microtiter plates, with a renewal of the media every 24 h. Following the 48 h biofilm growth, the groups were treated as described: Control: 200 µL of PBS in the dark; aBL alone: 200 µL of PBS (83 min in the dark) followed aBL (50 mW/cm^2^J/cm^2^); TCs followed by aBL: TCs incubated 83 min in the dark followed by aBL (50 mW/cm^2^–250 J/cm^2^); aBL followed by TCs incubation: 200 µL of PBS followed by aBL (50 mW/cm^2^–250 J/cm^2^) and after that, the addition of TCs (incubated 83 min in the dark). All groups were tested under the same conditions, and those without any irradiation served as dark control groups. After treatment, the biofilms were collected according to the method described in Section 2.7 and the CFU/mL was determined.

### 2.9. Measurement of Reactive Oxygen Species (ROS)

#### 2.9.1. Using DCFH-DA

The production of total ROS (such as singlet oxygen and hydroxyl radicals) induced by TCs and aBL, was measured using the general ROS probe, 2′,7′-dichlorofluorescein (DCFH-DA). The experiment was conducted either with planktonic or biofilm MRSA. Typically, the bacteria were cultured overnight in BHI broth at 37 °C with shaking. Cells were then harvested by centrifugation at 4000 rpm for 5 min and resuspended in PBS to a density of 10^8^ CFU/mL for planktonic experiment and 10^6^ CFU/mL for biofilm. For planktonic cultures, the microbial suspension or PBS was only transferred to 96-well plates while the biofilms grew for 48 h. After that, the samples were mixed with 10 µg/mL of TCs, and stained with 10 μM of DCFH-DA. The samples were irradiated with blue light at 50 mW/cm^2^ and collected every 20 min for analysis using a Microplate Spectrofluorometer (SPECTRAmax^®^, Molecular Devices, San Jose, CA, USA) according to probe wavelength of excitation and fluorescence emission (495/525 nm). The PBS with DCFH-DA served as control and the TCs/dark groups were generated under the same conditions without irradiation.

#### 2.9.2. Flow Cytometry Analysis of Reactive Oxygen Species (ROS) Production

Reactive oxygen species (ROS) production was assessed in methicillin-resistant *Staphylococcus aureus* (MRSA) planktonic cultures using a Celesta BD FACS Flow Cytometer (BD Biosciences, Woburn, MA, USA). Dihydrorhodamine 123 (DHR123) was used as a probe to quantify ROS levels. MRSA cultures were incubated with tetracyclines (TCs) at a concentration of 10 µg/mL for 83 min, after which DHR123 was added to the culture. Blue light exposure (50 mW/cm^2^) was administered for durations of 0 (dark), 30 (90 J/cm^2^), and 60 min (180 J/cm^2^). Following treatment, the bacterial cultures were analyzed using the flow cytometer system, with measurements taken for cell size and fluorescence intensity. A dark control was included by replicating the entire procedure without blue light exposure. Detailed information on the assay about gating strategy for flow cytometric analysis is depicted in Supplementary Figure S4.

## 3. Results

### 3.1. Spectroscopy

**Figure 1:**
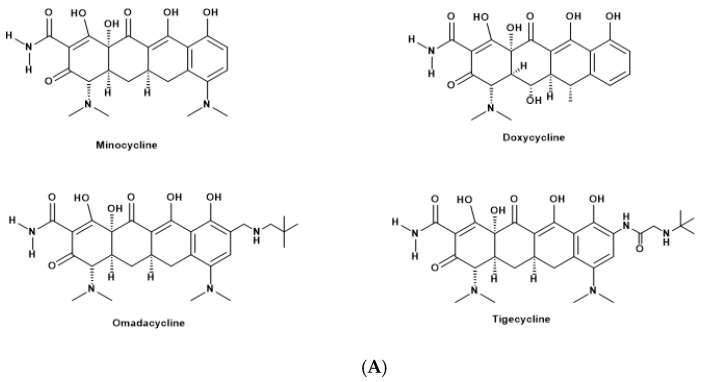

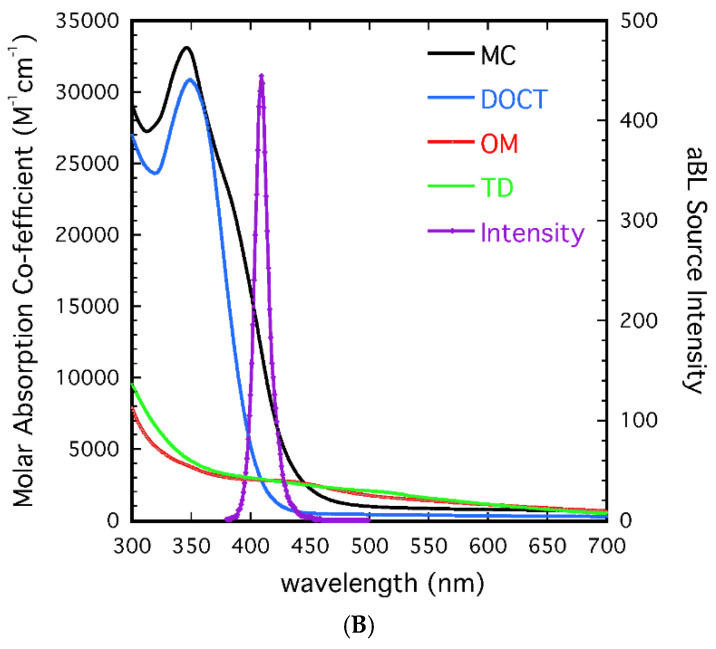
Chemical structure of (**A**) four tetracyclines: minocycline, doxycycline, omadacycline, and tigecycline. (**B**) Correlation between molar absorption coefficient and wavelength for each tetracycline in PBS and emission spectra of the blue light source (purple).

The chemical structures of the tetracyclines are shown in Figure 1A. MC, DOCT, OM, and TG are antibiotics belonging to the tetracycline class, each with distinct variations in their chemical structures. MC features a core tetracyclic ring system with a unique dimethylamino group at the C-7 position and hydroxyl groups at C-6 and C-12a. DOCT also has a tetracyclic core but is distinguished by its hydroxyl groups at C-5, C-6, and C-12a. TG, a glycylcycline, retains the tetracyclic structure but includes a modified side chain at C-9, incorporating a glycylamido group and a fluorine atom to enhance its efficacy and overcome resistance. OM maintains the tetracycline ring but has unique structural modifications, including a 7-(dimethylamino)-6-hydroxy group and a 3-(aminoacetyl)amino side chain, which broaden its antimicrobial spectrum and resistance profile [27]. We measured the absorption spectra of TCs from 200 to 800 nm and the emission spectra of the blue light source to analyze the optical properties in PBS (Figure 1). We correlated the molar absorption coefficient and wavelength of absorption for each tetracycline (Figure 1B). MC and DOTC displayed absorption spectra with broad absorption peaks at 350 nm in PBS, extending into the blue visible range. OM and TG lacked the 350 nm absorption band and showed weak absorption around 400 nm, likely due to modifications in their side chains. However, OM still exhibited absorption at 450 nm. The molar absorption coefficients at various wavelengths are detailed in Table 1. While MC exhibited the highest absorption coefficient at 410 nm, this did not necessarily correlate with microbicidal activity against in vitro biofilms.

### 3.2. Minimum Inhibitory Concentrations (MICs) of TCs (DOCT, MC, TG, OM) for MRSA, PA and E. coli

The MICs for all four tetracyclines (MC, DOCT, TG and OM) against MRSA, *E. coli*, and PA in planktonic culture were determined, as described, and shown in Table 2.

### 3.3. Antimicrobial Blue Light Dramatically Increases the Microbicidal Effects of TCs (DOCT, MC, TG, OM) on Biofilms of MRSA, PA and E. coli

After 48 h of incubation, mature monomicrobial biofilms of MRSA, PA, and *E.coli* were established, yielding 8.53 log10 CFU/mL, 9.35 log10 CFU/mL, and 7.45 log10 CFU/mL per well, respectively. Following treatment with aBL alone at 250 J/cm^2^ (50 mW/cm^2^), the log10 photoinactivation/reduction levels were −1.76 (*p* < 0.0001), −3.38 (*p* < 0.0001), and −1.93 (*p* < 0.0001) log10 CFU/mL for MRSA, PA, and *E. coli*, respectively. In the absence of aBL exposure, the tetracyclines did not demonstrate significant killing for any of the strains in biofilms. When biofilms were treated with various concentrations of tetracyclines (1, 2 and 4 µg/mL) in conjunction with 250 J/cm^2^ aBL, MRSA and PA underwent enhanced eradication with MC and DOCT, while enhanced bactericidal activity by aBL on *E*. *coli* was seen with OM and TG. As shown in Supplemental Material Figures S1–S3, the combination of 4 µg/mL of MC or DOCT with aBL resulted in log reductions of −2.72 log10 CFU/mL (*p* < 0.0001) and −3.83 log10 CFU/mL (*p* < 0.0001) for MRSA, and −5.39 log10 CFU/mL (*p* < 0.0001) and −5.25 log10 CFU/mL (*p* < 0.0001) for PA, respectively, thereby enhancing the effectiveness of aBL alone for both strains. For *E. coli* biofilms, the most effective results were obtained by combining 4 µg/mL of OM or TG with aBL, resulting in the eradication (reduction) of −3.82 log10 CFU/mL (*p* < 0.0001) and −3.68 log10 CFU/mL (*p* < 0.0001), respectively. This demonstrated an approximate 2-log enhancement in killing compared to aBL alone. These tetracycline-class drug concentrations would all be easily achievable when applied topically but would push the boundaries of achievable levels administered orally or systemically, as appropriate [28].

To summarize, the effectiveness of either a second-generation TC or a third- generation TC on bacterial biofilms was potentiated by at least one thousand-fold when combined with aBL treatment for the bacterial strains tested in vitro (Table 3). The data for the drugs and the three different bacterial species are depicted graphically in Supplementary Information Figures S1–S3.

### 3.4. Investigating the Ability of TCs (DOCT, MC, TG, OM) to Enhance ROS Levels by aBL

Noting some minimal absorption of 410 nm light by TCs and yet their substantial contribution to increased bactericidal activity, we investigated whether TCs might act as photosensitizers. We measured the production of reactive oxygen species (ROS) from TCs combined with blue light using the DCF-DA probe, which detects general ROS in the presence of MRSA. The acetylated form of 2′,7′-dichlorofluorescein (DCF-DA) is nonfluorescent until its acetate groups are cleaved by intracellular esterases, allowing for oxidation within the cell. The oxidation of this probe is indicated by increasing fluorescence signals. There is no fluorescence until it reacts with specific substrates, meaning that the fluorescence signal directly correlates with ROS production.

**Figure 2:**
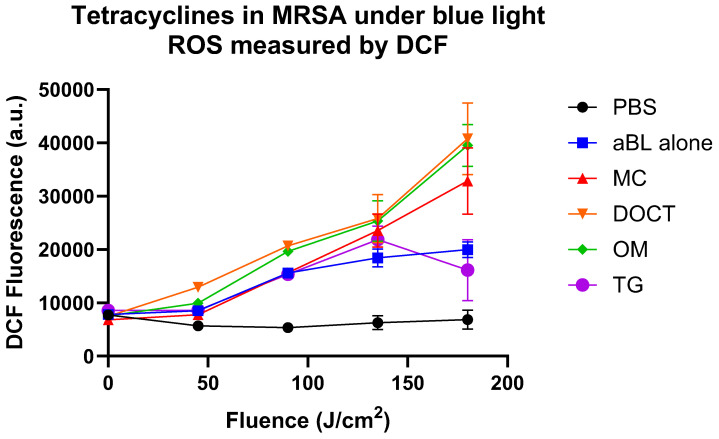
Measurement of reactive oxygen species levels using DCF-DA probe in MRSA planktonic culture treated with 10 µg/mL of TCs in combination with blue light.

**Figure 3:**
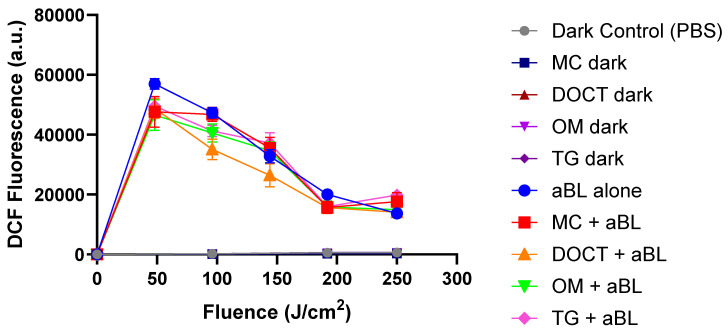
Measurement of reactive oxygen species levels using DCF-DA probe in MRSA biofilm treated with 10 µg/mL of TCs in combination with blue light.

In Figure 2, in planktonic culture, the probe demonstrated a consistent increase in fluorescence across all treatment conditions (TCs) upon exposure to blue light, accompanied by a corresponding rise in reactive oxygen species (ROS) production with extended exposure. We observed that MC, DOCT, and OM enhanced ROS production more effectively than blue light alone in planktonic bacteria. The combination of aBL with DOCT, MC, or OM led to a moderate increase in intracellular ROS, which aligned with the increased bacterial killing observed in Table 3. In contrast, TG resulted in lower ROS production, corresponding to reduced biofilm killing. Additionally, ROS levels were measured using the DCF-DA probe in MRSA biofilms. The results shown in Figure 3 indicated that ROS production in biofilms exposed to blue light alone was comparable to that in biofilms treated with tetracyclines and aBL, further suggesting that ROS production is not the primary mechanism responsible for the combined antimicrobial effects of tetracyclines and aBL.

**Figure 4:**
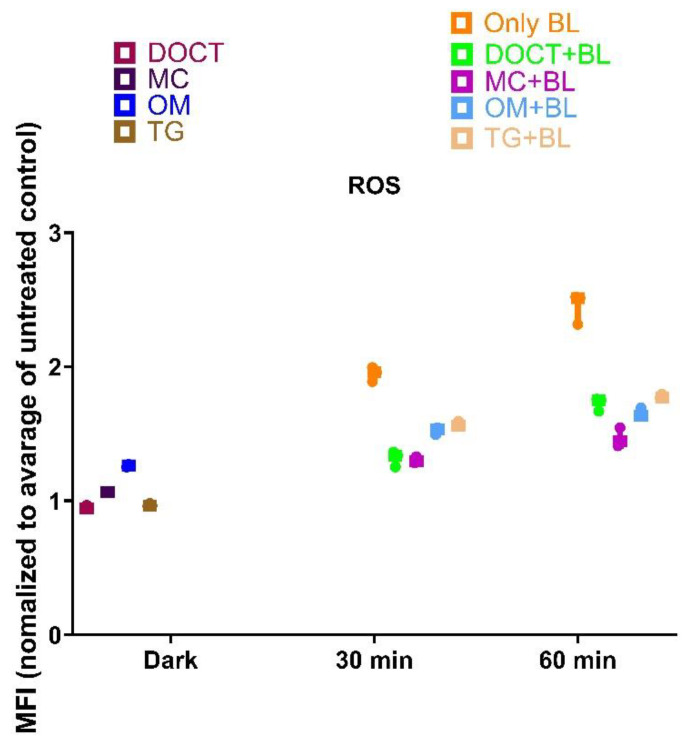
Measurement of reactive oxygen species by flow cytometer using dihydrorhodamine (DHR123) as a ROS probe in MRSA planktonic culture treated with 10 µg/mL of TCs in combination with blue light.

ROS production was also assessed using a flow cytometer with DHR123 as a probe (Figure 4), and the results revealed an increase in ROS levels upon blue light exposure relative to the non-irradiated controls. However, when the bacteria were incubated with tetracyclines (TCs) and subsequently exposed to blue light, no significant augmentation in ROS production was observed compared to blue light exposure alone. Although some ROS generation was detected, these results suggest that the mechanism through which tetracyclines enhance the antimicrobial blue light (aBL) effect is not predominantly mediated by an increase in ROS production.

### 3.5. Evaluation of TCs (DOCT, MC, TG, OM) as Potential Photosensitizers Under Blue Light Exposure of Biofilms

**Figure 5:**
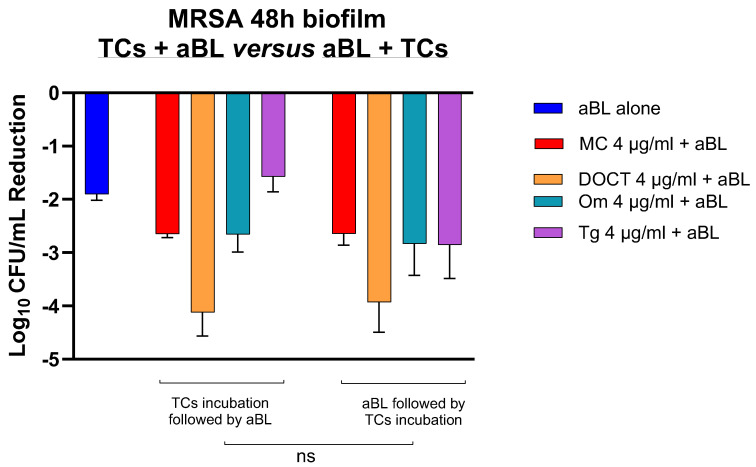
Bar graph illustrating the log10 colony-forming unit (CFU/g) reduction of 48 h of bacterial biofilms in MRSA after treatment with different conditions, procedure 1 (TCs incubation followed by aBL) and procedure 2 (aBL application followed by TCs incubation). The differences between untreated or treated biofilms were analyzed with a one-way ANOVA followed by Tukey’s multiple comparison tests: ns, not significant. The data for antibiotics alone in the dark are shown in Table 1.

After 48 h of biofilm incubation, mature monomicrobial biofilms of MRSA were established, reaching a concentration of 8.35 log10 CFU/mL per well. Two treatment procedures were compared. In procedure 1, the TCs were incubated for 83 min before exposure to aBL (50 mW/cm^2^, 250 J/cm^2^). In procedure 2, the MRSA were first irradiated with aBL, followed by the same incubation period with TCs. The results showed a reduction in MRSA levels after both timing procedures. Procedure 1 achieved reductions of 2.65 log10 CFU/mL (MC), 4.12 log10 CFU/mL (DOCT), 2.65 log10 CFU/mL (OM), and 1.57 log10 CFU/mL (TG). Procedure 2 resulted in comparable reductions of 2.64 log10 CFU/mL (MC), 3.92 log10 CFU/mL (DOCT), 2.83 log10 CFU/mL (OM), and 2.85 log10 CFU/mL (TG) for the same strain, with no significant difference between the two treatment protocols (Figure 5). If the TC, added after illuminating the bacteria has the same effect as when TC is added before illumination, the TC is unlikely to be acting primarily as a photosensitizer in the biofilm setting. We therefore cannot conclude from these data that the TCs are functioning as direct photodynamic photosensitizers on biofilm bacteria treated with blue light irradiation.

### 3.6. Evaluation to Determine the Effect of Post-aBL Incubation Time of TCs on Subsequent Bacterial Viability

**Figure 6:**
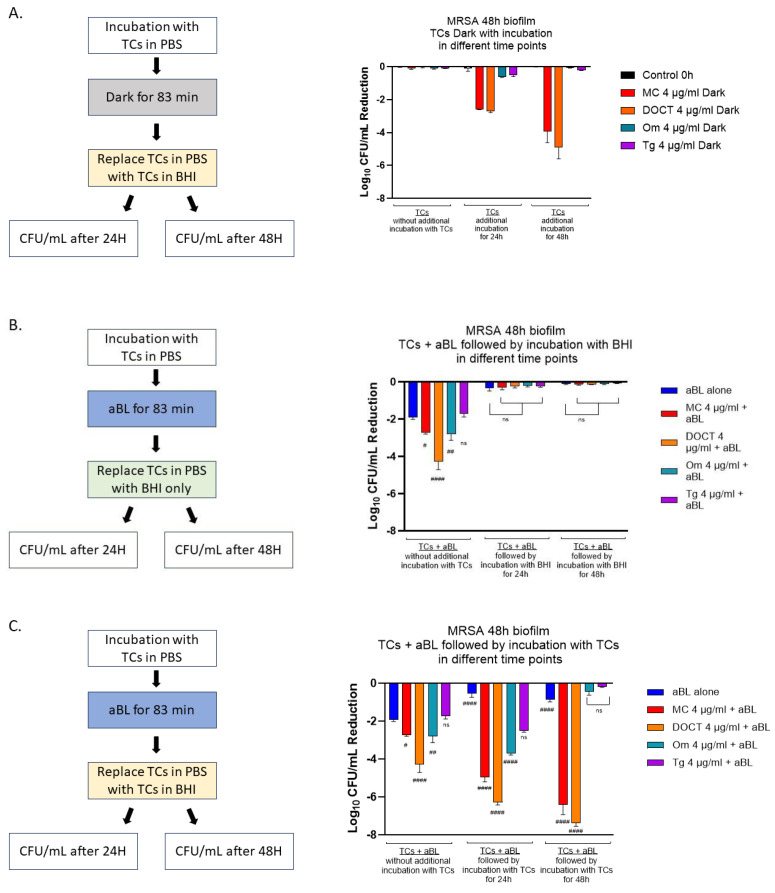
Comparation of post-effects of TC combined with aBL on MRSA biofilm at various time points—0 h, 24 h, and 48 h—both with and without TCs during these intervals. (**A**). Different time point incubation of TC without aBL (DARK). (**B**). Post-effect of TCs + aBL, followed by incubation with BHI only after 24 h and 48 h. (**C**). Post-effect of TCs + aBL, followed by incubation with TCs after 24 h and 48 h. The differences between aBL alone or aBL + TCs biofilms were analyzed with a one-way ANOVA followed by Tukey’s multiple comparison test: ns, not significant, #### *p* < 0.0001, ## *p* < 0.01, # *p* < 0.1, related to aBL.

We evaluated the post-irradiation effects of TC (4 µg/mL) after aBL on MRSA biofilm at various time points—0 h, 24 h, and 48 h post-aBL—both with and without TCs during these intervals. Immediately after aBL exposure, without any additional incubation time with TC, we observed reductions of 1.92 log10 CFU/mL (aBL alone), 2.72 log10 CFU/mL (MC), 4.26 log10 CFU/mL (DOCT), 2.79 log10 CFU/mL (OM), and 1.71 log10 CFU/mL (TG). After the initial treatment, TCs were removed and replaced with BHI only or BHI with respective TCs. When the TCs were replaced with BHI only, MRSA biofilm recovered after 24 h and 48 h, but no further killing was observed. However, when the MRSA biofilm was incubated with TCs for 24 h and 48 h following the first treatment (TCs + aBL), the effect of aBL was significantly enhanced. After 24 h of incubation with TCs, post-aBL treatment, the reductions increased to 4.93 log10 CFU/mL (MC), 6.26 log10 CFU/mL (DOCT), 3.68 log10 CFU/mL (OM), and 2.49 log10 CFU/mL (Tg). After 48 h of incubation with TCs, post-aBL treatment, the groups treated with MC and DOCT continued to show increased killing of MRSA, achieving reductions of 6.40 log10 CFU/mL (MC) and 7.37 log10 CFU/mL (DOCT). However, the MRSA biofilms treated with OM and TG began to recover after 48 h (Figure 6).

In other words, aBL significantly increased the activity of the second-generation tetracyclines on MRSA bacterial biofilms, when added post-irradiation. These effects were more pronounced with MC and DOTC, and not significant with the third-generation tetracyclines on MRSA.

These experiments demonstrate that the enhancing microbicidal effects of aBL on the tetracyclines could be overcome by re-feeding the in vitro biofilms with the protein-rich BHI media. However, after treatment with blue light, in the presence of either MC or DOCT, treatment of the in vitro 48 h biofilms with continued exposure to MC or DOCT for the following 24 and 48 h leads to a multi-log augmentation of the antimicrobial effects of both antibiotics.

## 4. Discussion

A conceptual breakthrough regarding the interaction of antibiotics with light came in 1983 with the observation by Hasan and Kahn that blue light interacted with tetracyclines to generate singlet oxygen, hypothesizing this as a contributor to antibacterial activity. Nitzan et al. described the effects of a blue light-photoactivated hematoporphyrin derivative on the viability of *Staphylococcus aureus* [29]. In 1986 it was reported that blue light (409 nm) was capable of interacting with porphyrins within *Propionibacterium acnes* bacteria, and similar observations were made with *Staphylococcus aureus* [29,30]. With these and other observations, the study of antibiotic interactions with light (synergism, antagonism, combinatorial effects was born.

Subsequently, the first and second generation tetracyclines were extensively studied with regard to their interactions with various wavelengths of light, with the objective of potentially pairing these two antimicrobial modalities together [11,18,19,22,23,31]. Virtually all of these prior papers involved experiments using planktonic cultures. There are far fewer data on antibiotic–aBL–biofilm interactions. However, the progressive development of AMR and the difficulty with treating biofilm infections strongly suggested to us the utility of such investigation [20,26,32].

In studies of the photochemical reactions stimulated by the exposure of tetracyclines to blue light, it has been demonstrated that this resulted in the generation of both hydroxyl radicals and singlet oxygen. Addition of potassium iodide at relatively high concentrations potentiated up to 5 logs of additional killing by light in planktonic culture [18,33]. Hamblin and Abrahamse [18,19] comprehensively reviewed this topic, further demonstrating that the tetracyclines can act as light-activated antibiotics, killing bacteria upon illumination, and do not absolutely require oxygen to produce photoactive effects. It was also hypothesized that residual tetracycline proximate to the bacterial 30S ribosome could then prevent bacterial regrowth after the withdrawal of illumination in vivo in infected mouse wounds, as was observed [18,31,34]. The other in vitro conclusions were entirely based on experiments performed with planktonic bacteria. Our in vitro data in biofilms do not support the conclusion that residual TC binding to the 30S ribosomal leads to a prolonged residual antimicrobial effect in biofilms, absent re-exposure to the drug. Our data suggest that the conditions of incubation are absolutely critical, i.e., re-feeding the biofilm cultures with protein-rich BHI media results in very little residual effect, but the effect of prior illumination indeed has an extraordinary multiplier effect when the topical TC is re-fed to the biofilm cultures. In other words, illumination of biofilms, either before or after the introduction of the TC results in dramatic, multi-log increases in TC-microbicidal effects, an effect essentially equal to illumination post-TC addition to the biofilm culture. This once again emphasizes the greater complexity of the effects of antimicrobial agents, be they antibiotics or light, when analyzing interactions in biofilms. Given the heterogeneous phenotypic nature of individual bacteria in biofilms, it is difficult to quantitate individual AMR mechanisms therein pre and post-treatment.

While advances in synthetic chemistry yielded newer and more potent tetracycline derivatives with broader antimicrobial spectra (reviewed [18,27], there developed an ever-expanding “tetracycline resistome” [18,35]. By 2009, there were already seven different tetracycline efflux pumps in just the first classification of these pumps [35]. Many more followed. It is therefore extremely difficult to de-convolute the changes in individual AMR mechanisms in biofilms both before and after illumination at different time points. We are only able to definitively state that aBL dramatically increases the antimicrobial effects of low levels of topical TC application either before or after introduction of the TC.

When blue light is applied, these antibiotics can, depending on their structure, generate reactive oxygen species (ROS),which can interact with adjacent chemical structures to interfere in their function [19,23,31,36,37,38]. This is more pronounced in the second generation TC’s, due to better absorption in the blue light wavelength range. One major shortcoming inherent to all light therapy is the fact that when the light is turned off, so does the photochemical antibacterial effect, no matter how powerful. The effects of that damage may play out over a prolonged period of time downstream. All of the previously referenced studies arrive at similar conclusions regarding the aforementioned findings. All of these studies largely involve planktonic culture methodology. There are limited data on photonic treatments of biofilm infections [26,32,39,40]. An additional shortcoming of aBL is poor depth of penetration.

In the in vitro biofilms, the tetracyclines did not demonstrate significant bactericidal effect for any of the three strains in the dark. With the addition of aBL, there were increased bactericidal effects particularly with MC and DOTC on MRSA and PA. A significant effect was also seen with OM and TG against *E. coli* in biofilms. One surprise, in which OM and TG interact with aBL in an unusually potent fashion, was against biofilms of *E. coli*, despite the poor light absorption of aBL by both these tetracyclines. We can only speculate at this point that this bactericidal effect was likely due to aBL having a damaging effect against structures in *E. coli* that function differently when detoxifying second generation versus third-generation tetracyclines. The ROS generation data of the drugs treated with aBL further suggested the inability of the tetracyclines to act as photosensitizers in biofilms.

In conclusion, with in vitro biofilm infections, the addition of antimicrobial blue light to the tetracyclines, acts at least additively, augmenting the activity to contribute to antimicrobial effectiveness. In the setting of wounds, especially chronic wounds which are a significant source of AMR, the combination of antimicrobial blue light and inexpensive, topical second-generation tetracyclines can provide yet another valuable tool to attack AMR development while speeding recovery from biofilm wound infections. In our prior studies, these largely in vitro studies were predictive for similar outcomes in our ex vivo porcine skin model and, in turn predictive of efficacy in our in vivo porcine wound model, the final step before human clinical studies [5]. Finally, the biofilm studies, in which the drugs alone were tested for bactericidal activity in 48 h in vitro biofilms, graphically demonstrate why clinical treatment of biofilm infections, even with the increased potency of the third-generation tetracyclines, can fall dramatically short of clinical expectations and MIC predictions. Our studies demonstrate that the inexpensive, generic second-generation tetracyclines, DOTC and MC are sufficiently active in concert with aBL so that there is little need to employ the far more expensive third-generation tetracyclines, TG and OM in the combined aBL treatment of biofilm wound infections. Augmentation of antimicrobial activity in biofilms by combining aBL with the tetracyclines demonstrates why aBL should be included in the therapeutic equation for the treatment of light-accessible wounds. It is inexpensive, safe, and has the potential to significantly improve antibiotic activities in the right setting.

## Figures and Tables

**Table 1:** TCs UV–Vis absorption parameters at different wavelengths.

Tetracyclines	ε (M^−1^·cm^−1^) (λ = 410 nm)
Minocycline (MC)	12,630
Doxycycline (DOCT)	2640
Omadacycline (OM)	2730
Tigecycline (TG)	2370

**Table 2:** Minimum Inhibitory Concentrations (MICs) of Tetracyclines Against MRSA, *E. coli*, and PA.

MS	MC (µg/mL)	DOCT (µg/mL)	OM (µg/mL)	TG (µg/mL)
MRSA	0.06	0.06	0.125	0.06
PA	8	16	64	8
*E. coli*	0.5	1	0.25	0.06

**Table 3:** aBL significantly increases sensitivity to TCs against MDR pathogens. Bacterial log10 CFU/mL reduction after the treatment of 48 h biofilm (MRSA, PA and *E. coli*) with the combination of TCs (4 µg/mL) and aBL at 50 mW/cm^2^–250 J/cm^2^. The differences between untreated or treated biofilms were analyzed with a one-way ANOVA followed by Tukey’s multiple comparison test: **** *p* < 0.0001, *** *p* < 0.001, ** *p* < 0.01, and * *p* < 0.1 compared to aBL alone, and ^####^ *p* < 0.0001 compared to dark control.

	MRSA Dark	MRSA aBL	PA Dark	PA aBL	*E. coli* Dark	*E. coli* aBL
aBL alone	-	−1.76 ^#####^	-	−3.38 ^#####^	-	−1.92 ^#####^
Minocycline	−0.13	−2.72 ****	0.06	−5.39 ****	−0.05	−2.35 **
Doxycycline	−0.26	−3.83 ****	−0.10	−5.24 ****	0.01	−2.50 ***
Omadacycline	−0.20	−2.38 *	−0.39	−4.42 *	−0.02	−3.83 ****
Tigecycline	0.015	−1.64	−0.89	−3.72	−0.03	−3.68 ****

## Data Availability

The data supporting the conclusions of our studies can be found in the Results section. Further details of the data can be obtained by contacting the corresponding author.

## Supplementary Materials

The following supporting information can be downloaded at: https://www.mdpi.com/article/10.3390/cells14030219/s1, Figure S1: Bar graph illustrating the log10 colony forming unit (CFU/mL) reduction of MRSA 48- hours biofilms after treatment with different concentrations (1, 2 and 4 µg/mL) of different TCs A-Minocycline; B-Doxycycline; C-Omadacycline; and D-Tigecycline) in combination with aBL (50 mW/cm^2^–250J/cm^2^) or without any light exposure (dark); Figure S2: Bar graph illustrating the log10 colony forming unit (CFU/mL) reduction of PA 48- hours biofilms after treatment with different concentrations (1, 2 and 4 µg/mL) of different TCs A-Minocycline; B-Doxycycline; C-Omadacycline; and D-Tigecycline) in combination with aBL (50 mW/cm^2^–250J/cm^2^) or without any light exposure (dark); Figure S3: Bar graph illustrating the log10 colony forming unit (CFU/mL) reduction of E.coli 48- hours biofilms after treatment with different concentrations (1, 2 and 4 µg/mL) of different TCs A-Minocycline; B-Doxycycline; C-Omadacycline; and D-Tigecycline) in combination with aBL (50 mW/cm^2^–250J/cm^2^) or without any light exposure (dark); Figure S4: Flow cytometer assay to measure ROS with the probe DHR123 after the treatment with TCs in different conditions: dark, 30 min (90 J/cm^2^) and 60 min (180 J/cm^2^) after aBL.
