# Supplementary material for: Antimicrobial Blue Light Reduces Human-Wound Pathogens’ Resistance to Tetracycline-Class Antibiotics in Biofilms
Source: Cells. 2025 Feb 4;14(3):219. doi: 10.3390/cells14030219 (PMC11817061; doi:10.3390/cells14030219)
Supplement: Supplementary file 1 [file cells-14-00219-s001.zip › cells-3412471-supplementary.pdf]

Supplementary Material

# Antimicrobial Blue Light Reduces Human-Wound Pathogens' Resistance to Tetracycline-Class Antibiotics in Biofilms

**Laisa Bonafim Negri [1,2,3,4], Sandeep Korupolu [1,3], William Farinelli [1,3], Alexis K. Jolly [5], Robert W. Redmond [1,3,4], Shifu Aggarwal [6,7], Laurence G. Rahme [6,7,8], Kristin H. Gilchrist [9,10], R. Rox Anderson [1,3,4] and Jeffrey A. Gelfand [1,2,3,4,11,*]**

[1] Wellman Center for Photomedicine, Massachusetts General Hospital (MGH), Boston, MA 02114, USA; lnegri@mgh.harvard.edu (L.B.N.); skorupolu@mgh.harvard.edu (S.K.); bfarinelli@mgb.org (W.F.); rredmond@mgh.harvard.edu (R.W.R.); rranderson@mgh.harvard.edu (R.R.A.)
[2] Vaccine & Immunotherapy Center, Division of Infectious Diseases, Massachusetts General Hospital (MGH), Boston, MA 02114, USA
[3] Department of Dermatology, Massachusetts General Hospital, Boston, MA 02114, USA
[4] Department of Dermatology, Harvard Medical School, Boston, MA 02114, USA
[5] School of Medicine, University of Edinburgh, Edinburgh EH16 4UX, UK; akjolly@mgh.harvard.edu
[6] Department of Surgery, Massachusetts General Hospital, Harvard Medical School, Boston, MA 02115, USA;
saggarwal5@mgh.harvard.edu (S.A.); lgrahme@mgb.org (L.G.R.)
[7] Department of Microbiology, Harvard Medical School, Boston, MA 02114, USA
[8] Shriners Hospitals for Children Boston, Boston, MA 02114, USA
[9] 4D Bio$^3$ Center for Biotechnology, Department of Radiology and Bioengineering, Uniformed Services
University of the Health Sciences, Bethesda, MD 20817, USA; kristin.gilchrist.ctr@usuhs.edu
[10] The Geneva Foundation, Tacoma, WA 98402, USA
[11] Department of Medicine, Harvard Medical School, Boston, MA 02115, USA
* Correspondence: jgelfand@mgh.harvard.edu; Tel. +1-617-803-8472

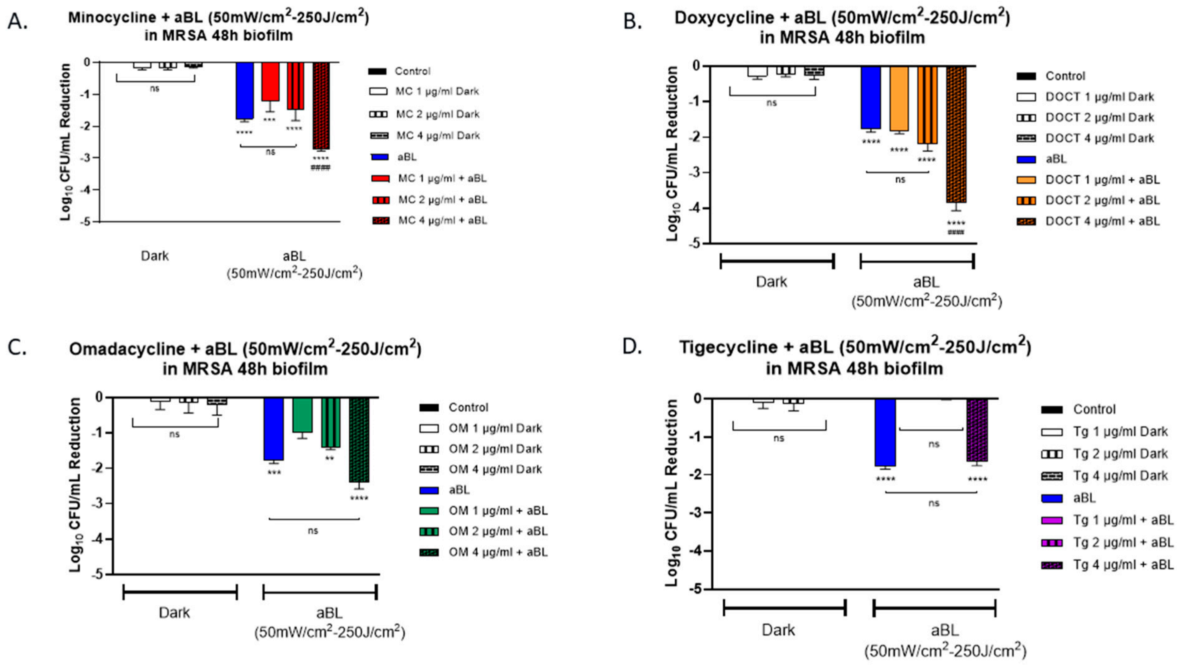


**Figure S1:** Bar graph illustrating the log10 colony forming unit (CFU/mL) reduction of MRSA 48- hours biofilms after treatment with different concentrations (1, 2 and 4 µg/mL) of different TCs A-Minocycline; B-Doxycycline; C-Omadacycline; and D-Tigecycline) in combination with aBL (50 mW/cm2-250J/$cm^2$) or without any light exposure (dark). The differences between untreated or treated biofilms were analyzed with a one-way ANOVA followed by Tukey's multiple comparison test: ns, not significant; ****$p<0.0001$, ***$p<0.001$, $p<0.01$, $p<0.1$ related to control. The differences between aBL alone or aBL + TCs biofilms were analyzed with a one-way ANOVA followed by Tukey's multiple comparison test: ns, not significant, ####$p<0.0001$, ###$p<0.001$, ##$p<0.01$, #$p<0.1$, related to aBL.

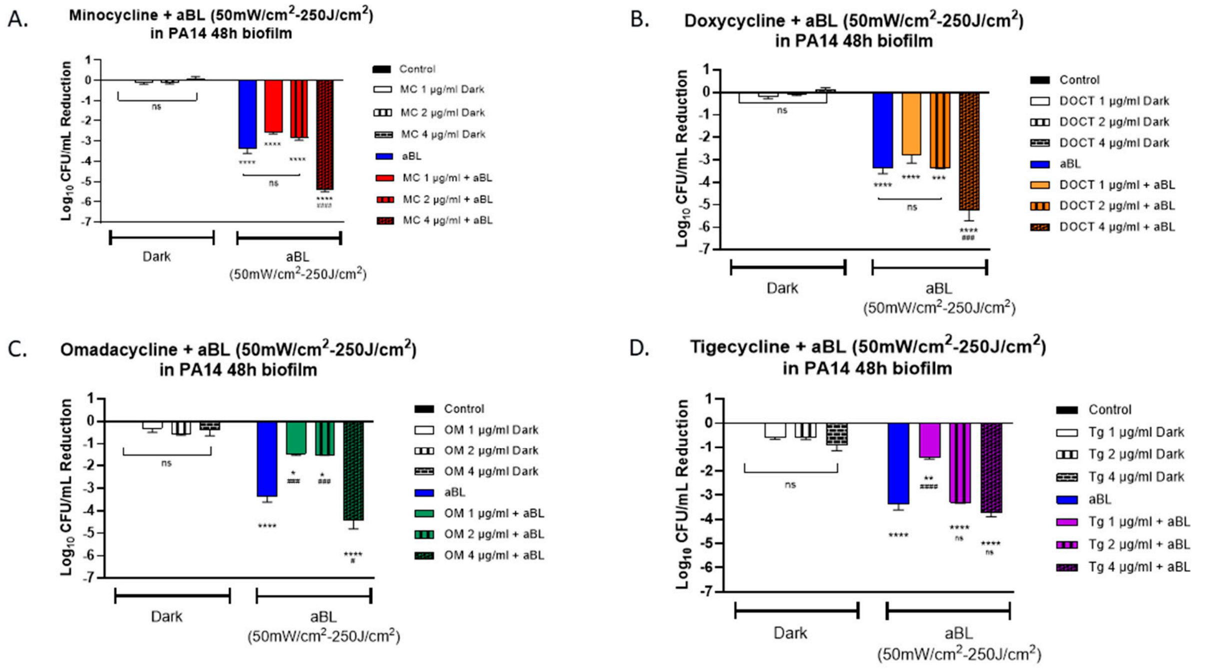


**Figure S2:** Bar graph illustrating the log10 colony forming unit (CFU/mL) reduction of PA 48-hours biofilms after treatment with different concentrations (1, 2 and 4 µg/mL) of different TCs A-Minocycline; B-Doxycycline; C-Omadacycline; and D-Tigecycline) in combination with aBL (50 mW/cm2-250J/$cm^2$) or without any light exposure (dark). The differences between untreated or treated biofilms were analyzed with a one-way ANOVA followed by Tukey's multiple comparison test: ns, not significant; ****$p<0.0001$, ***$p<0.001$, $p<0.01$, $p<0.1$ related to control. The differences between aBL alone or aBL + TCs biofilms were analyzed with a one-way ANOVA followed by Tukey's multiple comparison test: ns, not significant, ####$p<0.0001$, ###$p<0.001$, ##$p<0.01$, #$p<0.1$, related to aBL.

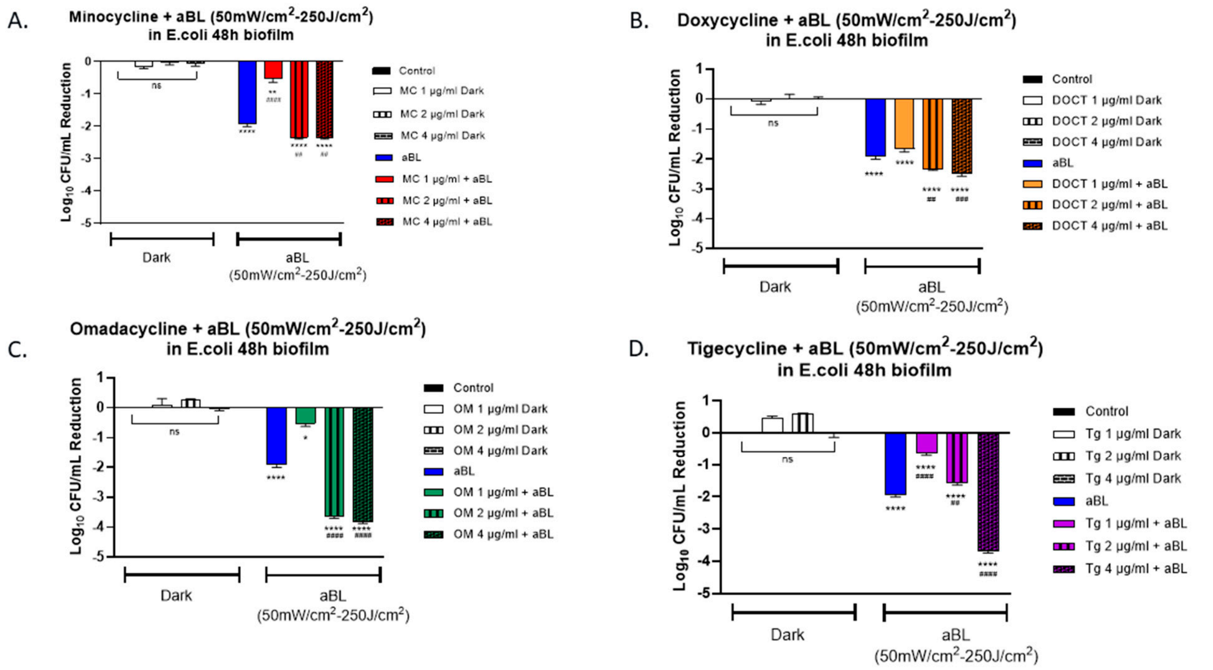


**Figure S3:** Bar graph illustrating the $\log_{10}$ colony forming unit (CFU/mL) reduction of *E.coli* 48-hours biofilms after treatment with different concentrations (1, 2 and 4 µg/mL) of different TCs A-Minocycline; B-Doxycycline; C-Omadacycline; and D-Tigecycline) in combination with aBL (50 mW/cm2-250J/$cm^2$) or without any light exposure (dark). The differences between untreated or treated biofilms were analyzed with a one-way ANOVA followed by Tukey's multiple comparison test: ns, not significant; ****p<0.0001, ***p<0.001, p<0.01, p<0.1 related to control. The differences between aBL alone or aBL + TCs biofilms were analyzed with a one-way ANOVA followed by Tukey's multiple comparison test: ns, not significant, ####p<0.0001, ###p<0.001, ##p<0.01, #p<0.1, related to aBL.

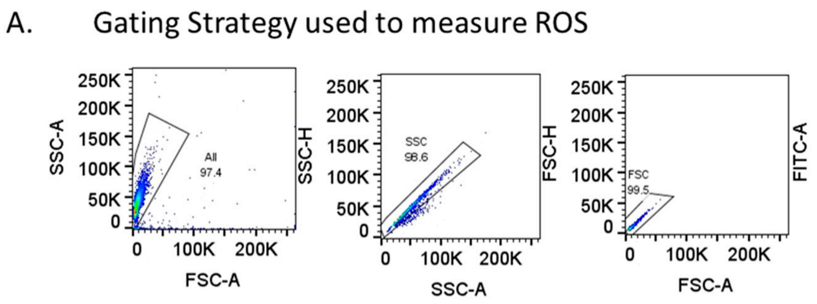

B. TCs in MRSA - DARK

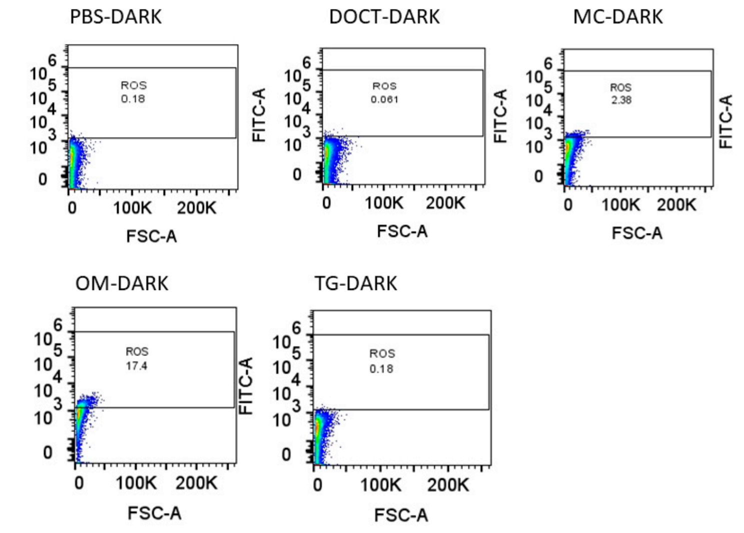


C. TCs + aBL in MRSA (90J/cm$^2$)

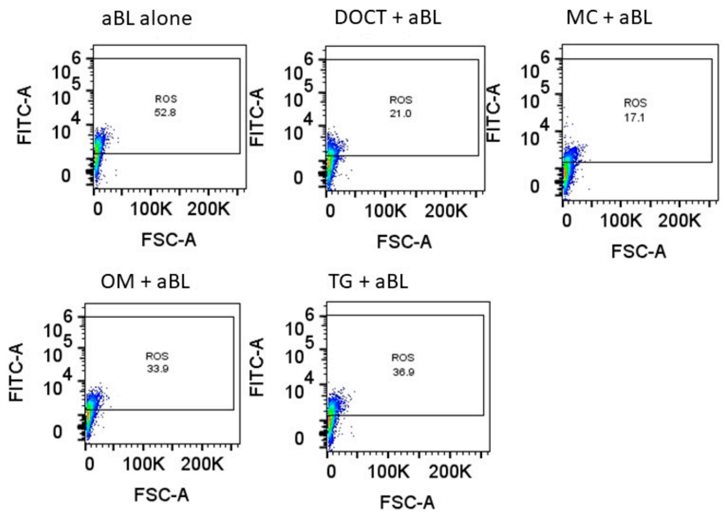

D. TCs + aBL in MRSA (180 J/cm$^2$)

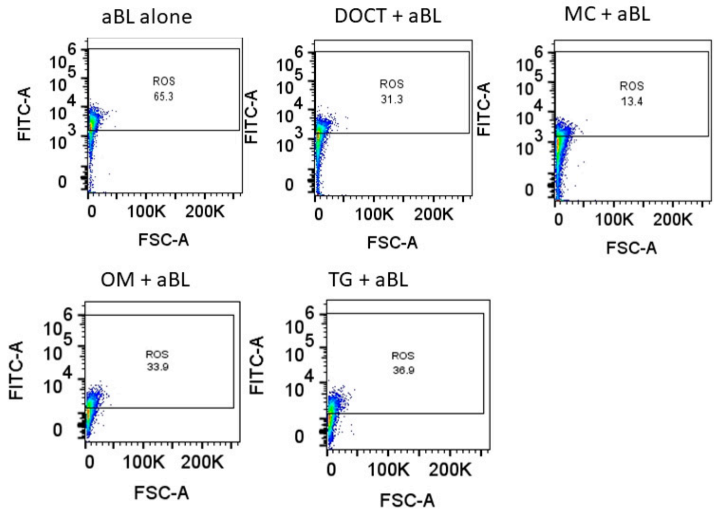


Figure S4: Flow cytometer assay to measure ROS with the probe DHR123 after the treatment with TCs in different conditions: dark, 30 min (90 J/cm$^2$) and 60 min (180 J/cm$^2$) after aBL. (A) The gating strategy used to measure ROS. Control and treated samples were stained with DHR123 and ROS gating was adjusted based on the control samples. Data were presented as mean fluorescent intensity (MFI). (B) ROS was measured in dark conditions (C) ROS was measured after 30 min after aBL (90 J/cm$^2$). (D) ROS was measured after 60 min after aBL (180 J/cm$^2$).
